# Rate, risk factors, and clinical outcomes of SARS-CoV-2 reinfection vs. primary infection in readmitted COVID-19 patients in Iran: a retrospective cohort study

**DOI:** 10.3389/fpubh.2024.1480805

**Published:** 2024-10-17

**Authors:** Mohammadamin Shahrbaf, Yousef Alimohamadi, Reza Yousefi Arfaei, Mahmood Salesi, Morteza Izadi, Mehdi Raei

**Affiliations:** ^1^Student Research Committee, Baqiyatallah University of Medical Sciences, Tehran, Iran; ^2^Health Research Center, Life Style Institute, Baqiyatallah University of Medical Sciences, Tehran, Iran; ^3^Chemical Injuries Research Center, Systems Biology and Poisonings Institute, Baqiyatallah University of Medical Sciences, Tehran, Iran

**Keywords:** COVID-19, SARS-CoV-2, reinfection, vaccination, prevention

## Abstract

**Background:**

Corona Virus Disease 2019 (COVID-19) has severely impacted global health, resulting in high morbidity and mortality, and overwhelming healthcare systems, particularly in Iran. Understanding reinfection is crucial as it has significant implications for immunity, public health strategies, and vaccine development. This study aims to identify rate and the risk factors associated with Severe Acute Respiratory Syndrome Coronavirus 2 (SARS-CoV-2) reinfection and compare the clinical course of initial infection versus reinfection in readmitted COVID-19 patients in Iran.

**Methods:**

This retrospective cohort study was conducted from January 2020 to the end of 2022 in five hospitals in Iran. The study compared demographic and clinical data, vaccination status, and clinical outcomes between patients with reinfection (defined as a positive PCR test for SARS-CoV-2 at least 90 days after the primary admission) and a control group (patients who had an initial confirmed SARS-CoV-2 infection but were not readmitted with a positive PCR test for SARS-CoV-2 at least 90 days after their primary infection). Risk factors for reinfection were evaluated using a regression model. Propensity score matching (PSM) was used to compare post-clinical and laboratory outcomes between the matched case and control groups.

**Results:**

Out of 31,245 patients, 153 (0.49%) experienced reinfections. The reinfection rate was significantly higher during B.1.617.2 and B.1.1.529 variant wave (*p* < 0.001). After multivariable regression analysis, incomplete vaccination status (OR: 1.68, 95% CI: 1.34–2.31, *p* = 0.021) and lack of booster vaccination (OR: 2.48, 95% CI: 1.96–3.65, *p* = 0.001) were the risk factors for reinfection. Furthermore, reinfection was associated with atypical COVID-19 symptoms, and shorter ICU and hospital stays (*p* < 0.001). The B.1.1.529 variant was significantly more common among reinfected patients (*p* < 0.001).

**Conclusion:**

SARS-CoV-2 reinfections are more frequently observed during waves of novel variants and are associated with a milder clinical course and shorter hospital stays. Full vaccination and booster doses can effectively reduce the risk of SARS-CoV-2 reinfections.

## 1 Introduction

Coronavirus Disease 2019 (COVID-19), caused by Severe Acute Respiratory Syndrome Coronavirus 2 (SARS-CoV-2), has profoundly impacted global health since its emergence in late 2019 (1–4). The pandemic has led to significant morbidity and mortality worldwide, overwhelming healthcare systems and disrupting economies (5, 6). With millions of confirmed cases and deaths, COVID-19 has highlighted the vulnerability of global health infrastructures to emerging infectious diseases (7, 8). The virus’s rapid spread and high transmissibility have necessitated unprecedented public health measures, including lockdowns, social distancing, and mass vaccination campaigns, to mitigate its impact (9–11).

The pandemic has unfolded in multiple waves, each characterized by different variants of the virus, varying levels of transmissibility, and distinct patterns of morbidity and mortality (12). These waves have placed immense pressure on healthcare resources and have been associated with significant fluctuations in case numbers and healthcare demand (13). The emergence of new variants, such as B.1.617.2 and B.1.1.529, has complicated efforts to control the pandemic, with each variant posing unique challenges in terms of transmissibility, vaccine effectiveness, and disease severity (14).

In Iran, the burden of COVID-19 has been substantial, with the country experiencing several waves of infection that have strained its healthcare system. High rates of infection and mortality have been reported, particularly during the peaks of the pandemic (15). The Iranian healthcare system has faced numerous challenges, including shortages of medical supplies, overwhelmed hospitals, and difficulties in implementing public health measures (16). Despite these challenges, efforts have been made to enhance testing, treatment, and vaccination to control the spread of the virus and reduce its impact on the population.

Despite extensive global research on COVID-19, understanding reinfection, particularly its clinical implications and associated variants, remains limited. Given the high burden of COVID-19 in Iran and the evolving nature of the virus, it is crucial to investigate SARS-CoV-2 reinfection in in this specific demographic. The aim of this study was to identify the rate and the risk factors associated with SARS-CoV-2 reinfection and compare the clinical course of initial infection versus reinfection in readmitted COVID-19 patients in Iran.

## 2 Materials and methods

### 2.1 Study design

This retrospective cohort study examines the rate, risk factors, and outcomes of SARS-CoV-2 reinfection in the Islamic Republic of Iran during all waves of the COVID-19 pandemic from January 2020 to the end of 2022. Data were collected from five referral hospitals in Tehran, Tabriz, Isfahan, Kerman, and Kermanshah. Sampling was conducted through a consecutive sampling method and the study population includes patients who were diagnosed with COVID-19 through a confirmed positive reverse transcription polymerase chain reaction (RT-PCR) test during their initial hospital admission and subsequently discharged. The inclusion criteria were readmission due to reinfection. Reinfection was defined as a subsequent positive PCR test for SARS-CoV-2 at least 90 days after the initial infection following readmission, based on the World Health Organization (WHO) criteria (17). Patients who did not have a documented reinfection were assumed to be the control group. The exclusion criteria included patients with a positive RT-PCR test for SARS-CoV-2 within 90 days of the initial infection, those hospitalized with clinical symptoms of COVID-19 without a follow-up RT-PCR test, those with incomplete medical records or missing relevant data, and those transferred to hospitals outside the study sites during their treatment.

### 2.2 Diagnosis of SARS-CoV-2

To determine the infection status, all patients were diagnosed with COVID-19 using RT-PCR tests performed on nasopharyngeal swab samples collected at the time of hospital admission. The RT-PCR assays targeted specific genes of SARS-CoV-2 and followed protocols approved by the national health authority in Iran and the WHO guidelines to ensure high sensitivity and specificity (18). When feasible, whole-genome sequencing was performed in reference laboratories on samples with sufficient viral load, and the variant classification was based on comparison with global reference data (19). In most cases, only the infection status (positive or negative) was available, and the variant determination was performed when appropriate testing resources were available.

### 2.3 Data collection

Data collection was conducted using a research-made checklist by the principal investigator. Data were collected from the integrated electronic health system of the hospitals and the medial record database. Demographic data, including age, sex, and the presence of underlying medical conditions such as hypertension (HTN), diabetes mellitus (DM), cardiovascular diseases, and cancer, were collected. The vaccination status and the status of receiving the vaccine booster dose of patients was recorded, and patients were classified into two groups: those who were fully vaccinated [received two doses of vaccine (20)] and those who were not fully vaccinated. The clinical symptoms were classified into two categories based on the chief complaint of patients at admission: common symptoms of SARS-CoV-2 infection, including cough, fever, shortness of breath, sore throat, fatigue, and myalgia; and less common symptoms, such as diarrhea, joint pain, and neurological disorders (21). Laboratory parameters, including white blood cell (WBC) count, Interleukin-6 (IL-6) levels, and C-reactive protein (CRP) levels, were also gathered. Moreover, the duration of their intensive care unit (ICU) and hospital stays, as well as their mortality during the admission period, were documented. When available, data on the variant of SARS-CoV-2 responsible for the infection were also collected.

### 2.4 Statistical analysis

Data were analyzed using SPSS version 25. After calculating the SARS-CoV-2 reinfection rate by dividing the number of reinfection cases by the total number of admitted patients, the quantitative data were presented as mean and standard deviation, while qualitative data were expressed as frequency and percentage. Comparisons were made between the reinfection group and the control (non-reinfection) group. The independent sample *t*-test and Chi-square test were used to compare quantitative and qualitative data, respectively. Risk factors for reinfection were evaluated through univariable and multivariable regression models. To ensure a balanced comparison between the clinical course and clinical outcomes of the reinfected and non-reinfected groups, propensity score matching (PSM) was conducted based on baseline demographic variables, including age, sex, and underlying disease. The matching was performed in a 1:1 ratio using R software. A *p-*value less than 0.05 considered as significant.

### 2.5 Ethical considerations

The study was conducted in accordance with ethical standards and guidelines to ensure the protection and confidentiality of patient information. Informed consent was waived by the ethics committee due to the retrospective nature of the study, which involved the analysis of existing medical records without direct patient interaction. To maintain confidentiality, all patient data were anonymized and stored securely. Access to the data was restricted to the research team members who were directly involved in the study. Approval was obtained from the Ethics Committee of Baqiyatallah University of Medical Sciences (Ethics code: IR.BMSU.REC.1400.159).

## 3 Results

### 3.1 Demographic data

A total of 31,245 patients with confirmed cases of SARS-CoV-2 infection from five hospitals in Tehran, Isfahan, Tabriz, Kerman, and Kermanshah were included in the final analysis to determine the rate of reinfection. Of these patients, 153 (0.49%) experienced reinfections based on the study criteria. Table 1 presents the status of reinfection in different hospitals and regions of Iran. The mean age of patients with reinfection was 54.5 ± 12.5 years, while the mean age in patients without confirmed reinfection was 62.3 ± 13.6 years (*p* < 0.001). Among the 153 patients, 128 (83.7%) were male and 25 (16.3%) were female. The sex distribution was significantly different between the reinfection group and the control group (*p* < 0.001). The demographic data are presented in Table 1.

**Table 1:** The rate of reinfection in different parts of Iran.

Hospital city	Number of COVID-19	Number of reinfections	Reinfection rate
Tehran (North of Iran)	13,179	68	0.51%
Isfahan (Central part of Iran)	7,102	18	0.25%
Tabriz (North east of Iran)	2,865	17	0.60%
Kerman (South west of Iran)	4,933	38	0.77%
Kermanshah (west part of Iran)	3,166	12	0.38%
Total number of patients	31,245	153	0.49%

### 3.2 Clinical, laboratory and outcome data

A total of 112 (73.2%) patients with reinfection reported at least one underlying condition such as diabetes, obesity, pulmonary disease, or cardiovascular disease (*p* = 0.041). In addition, 53 patients (34.6%) in the reinfection group had a full history of vaccination, while the number of fully vaccinated patients in patients without reinfection was 18,033 patients (57.9%, *p* < 0.001). Reinfection cases were more likely to present with atypical symptoms, whereas in patients without reinfection, COVID-19 generally manifested with typical symptoms (*p* < 0.001). In the assessment of laboratory values, patients with only primary infection had significant lower WBC count compared to patients in the reinfection group (8.5 ± 2 vs. 6.5 ± 2; *p* < 0.001). In addition, CRP and IL-6 levels were also significantly higher in primary SARS-CoV-2 infection (50 ± 20 vs. 30 ± 20; *p* < 0.001; and 40 ± 18 vs. 25 ± 15; *p* < 0.001, respectively). Primary SARS-CoV-2 infection resulted in longer ICU admissions compared to reinfections (5 ± 4 vs. 12 ± 10; *p* < 0.001). Also, patients with reinfection had shorter course of overall hospital stay (7 ± 6 vs. 16 ± 21; *p* < 0.001). Table 2 presents the clinical data of COVID-19 between the reinfection group and the control group.

**Table 2:** Clinical, laboratory and outcome data of patients with reinfection of SARS-CoV-2 and the control group.

Variable		Reinfection group (*n* = 153)	Control group (*n* = 31,092)	*p-*value
Age (year)		54.5 ± 12.5	62.3 ± 13.6	**<0.001**
Sex (%)	Male	128 (83.7)	19,277 (62)	**<0.001**
	Female	25 (16.3)	11,815 (38)	
Underlying disease (%)	Yes	112 (73.2)	20,210 (65)	**0.041**
	No	41 (26.8)	10,882 (35)	
Fully vaccinated (%)	Yes	53 (34.6)	18,033 (58)	**<0.001**
	No	100 (65.4)	13,059 (42)	
Vaccine booster (%)	Yes	12 (22.6)	9,214 (51.1)	**<0.001**
	No	41 (77.4)	8,819 (48.9)	
Symptoms (%)	Typical	112 (73.2)	29,227 (94)	**<0.001**
	Atypical	41 (26.8)	1865 (6)	
O_2_ saturation at admission (%)		85 ± 12.7	77 ± 18.7	**<0.001**
WBC (× 10^9^/L)		6.5 ± 2	8.5 ± 2	**<0.001**
CRP (mg/L)		30 ± 20	50 ± 25	**<0.001**
IL-6 (pg/mL)		25 ± 15	40 ± 18	**<0.001**
ICU stay (days)		5 ± 4	12 ± 10	**<0.001**
Hospital stay (days)		7 ± 6	16 ± 21	**<0.001**
Mortality during hospital stay (%)		3 (1.9)	2,685 (8.6)	**<0.001**

Bold *p* values are significant.

### 3.3 Risk factors of reinfection

In the univariable analysis, age was found to be a significant factor, with an odds ratio (OR) of 0.95 (95% CI: 0.93–0.97; *p* < 0.001). Males had significantly higher odds of reinfection, with an OR of 3.14 (95% CI: 2.51–4.02; *p* < 0.001). The presence of an underlying disease also showed a statistically significant association with reinfection risk, with an OR of 1.46 (95% CI: 1.11–1.93; *p* = 0.041). Vaccination status was strongly associated with reinfection risk, with an OR of 2.60 (95% CI: 2.06–3.41; *p* < 0.001). Additionally, vaccine booster status was the most significant predictor in the univariable analysis, with an OR of 3.57 (95% CI: 2.12–6.18; *p* < 0.001). After adjusting for potential confounders in the multivariable logistic regression model, vaccination remained a significant predictor of reduced reinfection risk (OR: 1.68, 95% CI: 1.34–2.31; *p* = 0.021). Also, vaccine booster status continued to be the most significant factor associated with reinfection, with those who did not receive a booster having an OR of 2.48 (95% CI: 1.96–3.65; *p* = 0.001). The results of the regression analysis are presented in Table 3.

**Table 3:** Risk factors of SARS-CoV-2 reinfection.

Variable	Univariable OR (95% CI)	*P-*value	Multivariable OR (95% CI)	*p-*value
Age	0.95 (0.93–0.97)	**<0.001**	0.99 (0.98–1.02)	0.749
Male gender	3.14 (2.51–4.02)	**<0.001**	1.18 (1.04–1.32)	0.432
Underlying disease	1.46 (1.11–1.93)	**0.041**	1.05 (1.01–1.28)	0.674
Not fully vaccinated	2.60 (2.06–3.41)	**<0.001**	1.68 (1.34–2.31)	**0.021**
Receive no vaccine booster	3.57 (2.12–6.18)	**<0.001**	2.48 (1.96–3.65)	**0.001**

Bold *p* values are significant.

### 3.4 Propensity score matching

After propensity score matching based on age, sex, and also underlying disease, atypical symptoms were significantly higher in patients with SARS-CoV-2 reinfection (26.8% vs. 6%; *p* < 0.001). In addition, the level of CRP in these patients was significantly lower than in patients in the control group (30 ± 20 vs. 40 ± 15; *p* < 0.001). Patients in the control group had a longer course of ICU stay (6 ± 3 vs. 5 ± 4; *p* = 0.014) and the hospital stay was significantly longer in control group (10 ± 5 vs. 7 ± 6; *p* < 0.001). There were no significant differences in terms of vaccination status, O_2_ saturation at admission, WBC, IL-6 and also mortality during hospital stay between the two groups (*p* = 0.702). Table 4 suggested the results after PSM.

**Table 4:** Clinical, laboratory and outcome data of patients with reinfection of SARS-CoV-2 and the control group after PSM.

Variable		Reinfection group (*n* = 153)	Control group (*n* = 153)	*p-*value
Age (year)		54.5 ± 12.5	54.8 ± 13	0.843
Gender (%)	Male	128 (83.7)	120 (78.4)	0.752
	Female	25 (16.3)	33 (21.6)	
Underlying disease (%)	Yes	112 (73.2)	113 (73.8)	0.994
	No	41 (26.8)	40 (26.2)	
Fully vaccinated (%)	Yes	53 (34.6)	72 (47)	0.348
	No	100 (65.4)	81 (53)	
Vaccine booster (%)	Yes	12 (22.6)	37 (51.4)	**0.002**
	No	41 (77.4)	35 (48.6)	
Symptoms (%)	Typical	112 (73.2)	43 (94)	**<0.001**
	Atypical	41 (26.8)	110 (6)	
O_2_ saturation at admission (%)		85 ± 12.7	83 ± 18.7	0.275
WBC (× 10^9^/L)		6.5 ± 2	6.8 ± 2.4	0.236
CRP (mg/L)		30 ± 20	40 ± 15	**<0.001**
IL-6 (pg/mL)		25 ± 15	28 ± 12	0.054
ICU stay (days)		5 ± 4	6 ± 3	**0.014**
Hospital stay (days)		7 ± 6	10 ± 5	**<0.001**
Mortality during hospital stay (%)		3 (1.9)	4 (2.6)	0.702

Bold *p* values are significant.

### 3.5 Different dates and COVID-19 waves

**Figure 1:**
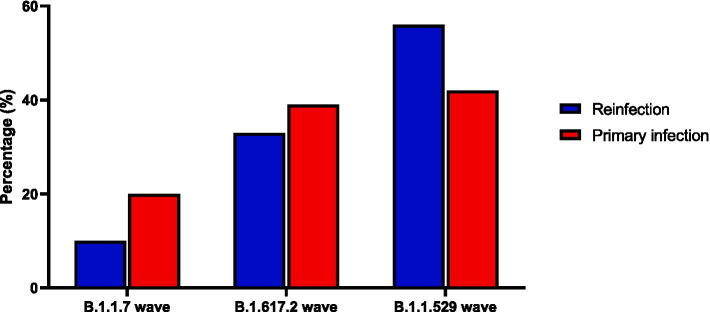
Distribution of SARS-CoV-2 variants among reinfection and control group across different waves.

Table 5 and Figure 1 show the distribution of SARS-CoV-2 variants in the reinfection and control groups over three different time periods corresponding to the B.1.1.7, B.1.617.2, and B.1.1.529 waves. During the B.1.1.7 wave (December 2020–June 2021), 10.5% of reinfection cases occurred compared to 19.6% of patients in the control group. In the B.1.617.2 wave (July 2021–November 2021), 33.4% of reinfections occurred compared to 38.8% of patients in the control group. During the B.1.1.529 wave (December 2021 – end of 2022), 56.1% of reinfections were recorded, compared to 41.6% of patients in the control group. The overall distribution of SARS-CoV-2 variants in the reinfected and control groups suggests a shift toward a higher likelihood of reinfection from B.1.1.7 to B.1.1.529 wave. The chi-squared test suggests that reinfections were more likely to occur during periods of new SARS-CoV-2 variants such as the B.1.1.529 and the B.1.617.2 variants (*p* < 0.001).

**Table 5:** Distribution of SARS-CoV-2 variants among reinfection and control group across different waves.

Time span	Reinfection (*n* = 153)	Control (*n* = 31,092)	*p-*value
December 2020–June 2021 (B.1.1.7 wave)	16 (10.5)	6,094 (19.6)	**<0.001**
July 2021–November 2021 (B.1.617.2 wave)	51 (33.4)	12,064 (38.8)	
December 2021 – End 2022 (B.1.1.529 wave)	86 (56.1)	12,934 (41.6)	

Bold *p* values are significant.

### 3.6 Different SARS-CoV-2 variants

The data for SARS-CoV-2 variants were present in 112 patients (73.2%) in the reinfection group and 4,364 patients (14%) in the control group. It was suggested that patients in the control group had significantly higher distribution of B1.1.7 variant while the reinfection group had more B1.1.529 variants. After propensity score matching based on age, sex, and also underlying disease, 53 patients remained in both groups. Same as the results before PSM, the B.1.617.2 and the B.1.1.529 variants were more prevalent in the reinfection group while the B.1.1.7 variant was more prevalent in the control group (*p* < 0.001). The results for SARS-CoV-2 variants in both groups are presented in Table 6.

**Table 6:** SARS-CoV-2 variants in reinfection and control group before and after PSM.

Variant	Before PSM			After PSM		
	Reinfection (*n* = 112)	Control (*n* = 4,364)	*p*-value	Reinfection (*n* = 53)	Control (*n* = 53)	*p-*value
B.1.1.7	12 (10.7)	2078 (47.6)	**<0.001**	6 (11.3)	26 (49)	**<0.001**
B.1.617.2	48 (42.9)	1,422 (32.6)		19 (35.8)	16 (30.2)	
B.1.1.529	52 (46.4)	864 (19.8)		28 (52.9)	11 (20.8)	

Bold *p* values are significant.

## 4 Discussion

The results of our study indicate that among the 0.5% of cases that met the WHO criteria for reinfection, these individuals had milder disease manifestations and a milder clinical course than those with primary infections. It has been suggested that reinfection is more pronounced in the new wave of SARS-CoV-2 variants and that full vaccination, especially booster vaccination, may be effective in preventing reinfection. It is noteworthy that the B.1.1.529 variant was more prevalent among those who had experienced reinfection. These findings contribute significantly to our understanding of virus dynamics and inform ongoing efforts in public health strategies, particularly in the context of emerging variants and vaccine efficacy.

It is crucial to distinguish between a reinfection with the SARS-CoV-2 virus that causes the disease and a return of symptoms. These two conditions can be challenging issues in clinical practice (22). The diagnosis of reinfection with SARS-CoV-2 is complex. Reinfection can sometimes be overreported based on clinical symptoms or radiological findings, and in some cases, PCR tests might be negative despite an active infection (23). In such scenarios, patients might experience a relapse of the original infection, or they could have other diseases with symptoms similar to SARS-CoV-2, such as influenza or respiratory syncytial virus (24). In addition, many individuals experiencing reinfection may not seek medical attention or may only visit outpatient clinics, thus not being captured in hospital-based data (25). The current study assumed a reinfection rate of 0.5%. This result is consistent with previous studies in the current literature. In the meta-analysis of 23 studies in 2023, the reinfection rate ranged from 0.1 to 6.8% (26). In addition, in a recent meta-analysis in 2024, the pooled rate of reinfection in 55 studies was estimated to be 0.94% (27). The observed difference in the reported rate of reinfection may be mainly due to differences in patient selection criteria. In our study, we made a concerted effort to carefully select patients in a manner that minimized the likelihood of false positives while adhering to WHO guidelines.

Our results align with previous studies suggesting that reinfections generally present with less severe outcomes compared to primary infections (28). The observation that cases of reinfection had shorter hospital and ICU stays is consistent with other reports indicating that immune responses from prior infections or vaccinations might reduce the severity of subsequent infections (29). Furthermore, the discrepancy in the predominant SARS-CoV-2 variants between reinfection and primary infection cases highlights the virus’s evolving nature and its impact on disease presentation (30). The lower frequency of full vaccination in reinfected individuals compared to the non-reinfected group also underscores the need for continued research into the effectiveness of current vaccines against emerging variants (31). Although this difference was not statistically significant following PSM, it remains critical to continue evaluating vaccine efficacy, particularly in the context of emerging variants. The effectiveness of vaccines against variants has been shown to diminish over time, necessitating booster doses to maintain protective immunity (32). Our study’s findings are consistent with the literature, which indicates that while vaccines remain effective in preventing severe disease, the evolving nature of SARS-CoV-2 variants calls for regular updates to vaccination protocols and booster recommendations (33).

The distribution of SARS-CoV-2 variants in the reinfection and control groups highlighted a clear trend toward a higher proportion of reinfections during waves dominated by newer variants. Reinfections were less frequent during the B.1.1.7 wave. This trend shifted significantly with the emergence of the B.1.617.2 variant and became even more pronounced during the B.1.1.529 wave. The increasing reinfection rate in later waves is consistent with the immune-evading properties of newer variants such as B.1.1.529, which have been shown to have higher transmissibility and reduced vaccine efficacy (34). Studies have shown that B.1.1.529, with its numerous spike protein mutations, is more adept at escaping both natural immunity and vaccine-induced immunity, leading to increased reinfection rates (35). Our analysis also demonstrated that individuals who received a vaccine booster were significantly less likely to experience reinfection, even during the B.1.1.529 wave, highlighting the positive effect of booster doses in enhancing protection against immune-evading variants. This is further supported by studies showing that booster doses can restore vaccine efficacy against variants such as B.1.1.529 (36).

The strength of our study lies in its large, representative sample of over 30,000 confirmed SARS-CoV-2 infection cases from five major hospitals in different regions of Iran, ensuring a robust analysis of reinfection patterns and outcomes. By adhering to WHO criteria for reinfection and focusing on hospitalized patients with confirmed PCR results, we ensured high diagnostic accuracy. The study’s findings of greater susceptibility to reinfection during the B.1.1.529 wave and the protective effect of booster doses are consistent with global research and underscore the importance of booster vaccination against immune-evading variants. In addition, the inclusion of regional data highlights the impact of healthcare disparities in Iran, where differences in access to resources may influence hospitalization and outcomes.

It is important to acknowledge the limitations of our study, despite its robust design. The first limitation is the reliance on PCR testing as the sole method for confirming SARS-CoV-2 infection, which may not capture all cases, particularly those with low viral loads where PCR sensitivity might be reduced. This could potentially lead to an underestimation of reinfection rates. However, by focusing on positive PCR results, we ensured diagnostic certainty and accuracy in identifying true reinfection cases. Another significant challenge encountered in this study was the limited access to diagnostic kits for SARS-CoV-2 variant identification in Iran. Due to sanctions, these kits were frequently unavailable or in short supply, which constrained our capacity to accurately identify specific variants. Additionally, many of the reinfection cases occurred after the introduction of variant-specific diagnostic kits, whereas a large portion of the control cases were from a period when new variants had not yet been identified. Consequently, variant data, particularly in the control group, may have been underrepresented. To address this imbalance and ensure a robust comparison between the two groups, we applied PSM. Another limitation is the lack of detailed data on treatment scenarios. Although there are established guidelines for COVID-19 treatment, the treatment protocols changed over the pandemic time (37). In addition, the administration of management strategies varied depending on the availability of pharmaceuticals and the limitation of resources in different cities and medical centers. The last limitation of our study is the lack of extended follow-up data. Although reinfections generally present with milder symptoms, they may be associated with severe long-term complications such as stroke, myocardial infarction, deep vein thrombosis, or pulmonary embolism (38).

This study recommends prioritizing revaccination campaigns to improve protection against reinfection with SARS-CoV-2, especially in the face of emerging immune-evading variants such as B.1.1.529. Public health strategies should be regularly updated to reflect the evolving nature of the virus, and efforts must be made to improve access to variant-specific diagnostic tools in resource-limited settings. In addition, future research should focus on longer-term follow-up to assess potential complications of reinfection, such as cardiovascular events, to provide a more comprehensive understanding of the long-term effects of SARS-CoV-2.

## 5 Conclusion

SARS-CoV-2 reinfection generally exhibited milder symptoms and shorter hospital stays than primary infections. Novel SARS-CoV-2 variants were more common among reinfected individuals. Although vaccination can help prevent reinfection, the complex relationship between vaccination and reinfection highlights the need for further research. Future studies are needed to assess the long-term complications of SARS-CoV-2 reinfection.

## Data Availability

The raw data supporting the conclusions of this article will be made available by the authors, without undue reservation.

## References

[1] Shahrbaf MA Tabary M Khaheshi I. Cardiovascular considerations of Remdesivir and Favipiravir in the treatment of COVID-19. Cardiovasc Haematol Disord Drug Targets. (2021) 21:88–90. doi: 10.2174/1871529X21666210812103535, PMID: 34387172

[2] Shahrbaf MA Tabary M Khaheshi I. The right ventricle in COVID-19 patients. Eur Heart J. (2021) 42:559–60. doi: 10.1093/eurheartj/ehaa832, PMID: 33206948 PMC7717190

[3] Barekat M Shahrbaf MA Rahi K Vosough M. Hypertension in COVID-19, a risk factor for infection or a late consequence? Cell J. (2022) 24:424. doi: 10.22074/cellj.2022.8487, PMID: 36043411 PMC9428472

[4] Robat-Jazi B Ghorban K Gholami M Samizadeh E Aghazadeh Z Shahrbaf MA. β-D-mannuronic acid (M2000) and inflammatory cytokines in COVID-19; an in vitro study. Iran J Allergy Asthma Immunol. (2022) 21:677–86. doi: 10.18502/ijaai.v21i6.11528, PMID: 36640059

[5] Shahrbaf MA Hassan M Vosough M. COVID-19 and hygiene hypothesis: increment of the inflammatory bowel diseases in next generation? Expert Rev Gastroenterol Hepatol. (2022) 16:1–3. doi: 10.1080/17474124.2022.2020647 34919489

[6] Masoumbeigi H Mirshafiee A Ghanizadeh G Raei M Saffarri M Arfaei RY. Evaluation of the effect of educational interventions on knowledge, attitude, and practice against COVID-19 in a residential complex in Tehran: a prospective cross-sectional study. Med J Islam Repub Iran. (2023) 37:50. doi: 10.47176/mjiri.37.50, PMID: 37426480 PMC10329504

[7] Tehrani S Fekri S Demirci H Nourizadeh AM Kashefizadeh A Shahrbaf MA. Coincidence of candida endophthalmitis, and aspergillus and pneumocystis jirovecii pneumonia in a COVID-19 patient: case report. Ocul Immunol Inflamm. (2023) 31:1291–4. doi: 10.1080/09273948.2023.2188224, PMID: 36952481

[8] Zarrabi M Shahrbaf MA Nouri M Shekari F Hosseini S-E Hashemian S-MR. Allogenic mesenchymal stromal cells and their extracellular vesicles in COVID-19 induced ARDS: a randomized controlled trial. Stem Cell Res Ther. (2023) 14:169. doi: 10.1186/s13287-023-03402-8 37365605 PMC10294333

[9] Raei M Shahrbaf MA Salaree MM Yaghoubi M Parandeh A. Prevalence and predictors of burnout among nurses during the COVID-19 pandemic: a survey in teaching hospitals. Work. (2023) 77:1049–57. doi: 10.3233/WOR-220001 37781833

[10] Saberi-Hamedani M Amiri P Keramatinia A Shahrbaf MA Shekarriz-Foumani R. The prediction of suicide ideation based on perceived social support, personality traits, and meaning of life in medical students during COVID-19 pandemic: a cross-sectional study. Int J Body Mind Cult. (2023) 10:2345–5802. doi: 10.22122/ijbmc.vi.485

[11] Shahrbaf MA Nasr DS Langroudi ZT. COVID-19 and health promoting hospitals in Iran; what do we stand? International journal of preventive medicine. Int J Prev Med. (2022) 13:13–125. doi: 10.4103/ijpvm.ijpvm_492_21 36452476 PMC9704482

[12] El-Shabasy RM Nayel MA Taher MM Abdelmonem R Shoueir KR Kenawy ER. Three waves changes, new variant strains, and vaccination effect against COVID-19 pandemic. Int J Biol Macromol. (2022) 204:161–8. doi: 10.1016/j.ijbiomac.2022.01.118, PMID: 35074332 PMC8782737

[13] Moynihan R Sanders S Michaleff ZA Scott AM Clark J To EJ. Impact of COVID-19 pandemic on utilisation of healthcare services: a systematic review. BMJ Open. (2021) 11:e045343. doi: 10.1136/bmjopen-2020-045343, PMID: 33727273 PMC7969768

[14] Dhama K Nainu F Frediansyah A Yatoo MI Mohapatra RK Chakraborty S. Global emerging omicron variant of SARS-CoV-2: impacts, challenges and strategies. J Infect Public Health. (2023) 16:4–14. doi: 10.1016/j.jiph.2022.11.024, PMID: 36446204 PMC9675435

[15] Heidari M Sayfouri N Jafari H. Consecutive waves of COVID-19 in Iran: various dimensions and probable causes. Disaster Med Public Health Prep. (2022) 17:e136. doi: 10.1017/dmp.2022.45 35152937 PMC9509792

[16] Khankeh H Farrokhi M Roudini J Pourvakhshoori N Ahmadi S Abbasabadi-Arab M. Challenges to manage pandemic of coronavirus disease (COVID-19) in Iran with a special situation: a qualitative multi-method study. BMC Public Health. (2021) 21:1919. doi: 10.1186/s12889-021-11973-5, PMID: 34686165 PMC8532398

[17] Chisale MRO Sinyiza FW Kaseka PU Chimbatata CS Mbakaya BC Wu TJ. Coronavirus disease 2019 (COVID-19) reinfection rates in Malawi: a possible tool to guide vaccine prioritisation and immunisation policies. Vaccines. (2023) 11:1185. doi: 10.3390/vaccines11071185 37515002 PMC10383452

[18] Dip SD Sarkar SL Setu MAA Das PK Pramanik MHA Alam A. Evaluation of RT-PCR assays for detection of SARS-CoV-2 variants of concern. Sci Rep. (2023) 13:2342. doi: 10.1038/s41598-023-28275-y, PMID: 36759632 PMC9910272

[19] Ntagereka PB Oyola SO Baenyi SP Rono GK Birindwa AB Shukuru DW. Whole-genome sequencing of SARS-CoV-2 reveals diverse mutations in circulating alpha and Delta variants during the first, second, and third waves of COVID-19 in south Kivu, east of the Democratic Republic of the Congo. Int J Infect Dis. (2022) 122:136–43. doi: 10.1016/j.ijid.2022.05.041, PMID: 35598737 PMC9119719

[20] Seo WJ Kang J Kang HK Park SH Koo HK Park HK. Impact of prior vaccination on clinical outcomes of patients with COVID-19. Emerg Microbes Infect. (2022) 11:1316–24. doi: 10.1080/22221751.2022.2069516, PMID: 35465831 PMC9132471

[21] Adhikari SP Meng S Wu Y-J Mao Y-P Ye R-X Wang Q-Z. Epidemiology, causes, clinical manifestation and diagnosis, prevention and control of coronavirus disease (COVID-19) during the early outbreak period: a scoping review. Infect Dis Poverty. (2020) 9:29. doi: 10.1186/s40249-020-00646-x, PMID: 32183901 PMC7079521

[22] Raveendran AV Jayadevan R Sashidharan S. Long COVID: an overview. Diabetes Metab Syndr. (2021) 15:869–75. doi: 10.1016/j.dsx.2021.04.007, PMID: 33892403 PMC8056514

[23] Raveendran AV. COVID-19 re-infection: diagnostic challenges and proposed diagnostic criteria. Diabetes Metab Syndr. (2021) 15:645–8. doi: 10.1016/j.dsx.2021.02.007, PMID: 33663969 PMC7877869

[24] Phan T Tran NYK Gottlieb T Siarakas S McKew G. Evaluation of the influenza and respiratory syncytial virus (RSV) targets in the AusDiagnostics SARS-CoV-2, influenza and RSV 8-well assay: sample pooling increases testing throughput. Pathology. (2022) 54:466–71. doi: 10.1016/j.pathol.2022.02.002, PMID: 35461715 PMC9021007

[25] Azam M Pribadi FS Rahadian A Saefurrohim MZ Dharmawan Y Fibriana AI. Incidence of COVID-19 reinfection: an analysis of outpatient-based data in the United States of America. medRxiv. (2021) 2021:7206. doi: 10.1101/2021.12.07.21267206

[26] Nguyen NN Nguyen YN Hoang VT Million M Gautret P. SARS-CoV-2 reinfection and severity of the disease: a systematic review and Meta-analysis. Viruses. (2023) 15:967. doi: 10.3390/v15040967, PMID: 37112949 PMC10145185

[27] Chen Y Zhu W Han X Chen M Li X Huang H. How does the SARS-CoV-2 reinfection rate change over time? The global evidence from systematic review and meta-analysis. BMC Infect Dis. (2024) 24:339. doi: 10.1186/s12879-024-09225-z, PMID: 38515023 PMC10956270

[28] Deng J Ma Y Liu Q Du M Liu M Liu J. Severity and outcomes of SARS-CoV-2 reinfection compared with primary infection: a systematic review and Meta-analysis. Int J Environ Res Public Health. (2023) 20:3335. doi: 10.3390/ijerph20043335 36834029 PMC9961977

[29] de La Vega MA Polychronopoulou E Xiii A Ding Z Chen T Liu Q. SARS-CoV-2 infection-induced immunity reduces rates of reinfection and hospitalization caused by the Delta or omicron variants. Emerg Microbes Infect. (2023) 12:e2169198. doi: 10.1080/22221751.2023.2169198, PMID: 36655944 PMC9980403

[30] Manirambona E Okesanya OJ Olaleke NO Oso TA Lucero-Prisno DE. Evolution and implications of SARS-CoV-2 variants in the post-pandemic era. Discover Public Health. (2024) 21:16. doi: 10.1186/s12982-024-00140-x

[31] Gómez-Gonzales W Chihuantito-Abal LA Gamarra-Bustillos C Morón-Valenzuela J Zavaleta-Oliver J Gomez-Livias M. Risk factors contributing to reinfection by SARS-CoV-2: a systematic review. Adv Respir Med. (2023) 91:560–70. doi: 10.3390/arm91060041, PMID: 38131876 PMC10740414

[32] Dadras O SeyedAlinaghi S Karimi A Shojaei A Amiri A Mahdiabadi S. COVID-19 Vaccines' protection over time and the need for booster doses; a systematic review. Arch Acad Emerg Med. (2022) 10:e53. doi: 10.22037/aaem.v10i1.1582, PMID: 36033989 PMC9397599

[33] Hogan AB Doohan P Wu SL Mesa DO Toor J Watson OJ. Estimating long-term vaccine effectiveness against SARS-CoV-2 variants: a model-based approach. Nat Commun. (2023) 14:4325. doi: 10.1038/s41467-023-39736-3, PMID: 37468463 PMC10356855

[34] He X Hong W Pan X Lu G Wei X. SARS-CoV-2 omicron variant: characteristics and prevention. MedComm. (2021) 2:838–45. doi: 10.1002/mco2.110 34957469 PMC8693031

[35] Pulliam JRC van Schalkwyk C Govender N von Gottberg A Cohen C Groome MJ. Increased risk of SARS-CoV-2 reinfection associated with emergence of omicron in South Africa. Science. (2022) 376:abn4947. doi: 10.1126/science.abn4947 PMC8995029 35289632

[36] Bar-On YM Goldberg Y Mandel M Bodenheimer O Amir O Freedman L. Protection by a fourth dose of BNT162b2 against omicron in Israel. N Engl J Med. (2022) 386:1712–20. doi: 10.1056/NEJMoa2201570 35381126 PMC9006780

[37] Wu Y Feng X Gong M Han J Jiao Y Li S. Evolution and major changes of the diagnosis and treatment protocol for COVID-19 patients in China 2020–2023. Health Care Sci. (2023) 2:135–52. doi: 10.1002/hcs2.45, PMID: 38939112 PMC11080729

[38] Bowe B Xie Y Al-Aly Z. Acute and postacute sequelae associated with SARS-CoV-2 reinfection. Nat Med. (2022) 28:2398–405. doi: 10.1038/s41591-022-02051-3, PMID: 36357676 PMC9671810

